# Sonographic diagnosis of carpal tunnel syndrome

**DOI:** 10.1590/0100-3984.2015.48.6e2

**Published:** 2015

**Authors:** Rodrigo O. C. Aguiar

**Affiliations:** 1 Universidade Federal do Paraná (UFPR) and DAPI - Diagnóstico Avançado por Imagem, Curitiba, PR, Brazil. E-mail: aguiar.rodrigo@gmail.com.

Carpal tunnel syndrome (CTS) is the most common compressive neuropathy of the upper extremity, affecting 2% to 5% of the population, usually involving compression of the median nerve in the wrist region^(1)^. The prevalence is higher in women than in men, and most commonly the disease onset occurs in the age range between 30 and 60 years. In older patients, the female/male ratio is up to 4:1, and the dominant hand is generally most affected; but bilaterality may occur in up to 50% of cases^(2)^, making the comparison of images with the contralateral side more difficult, considering that nerve abnormalities may present previously to symptoms onset. The incidence of CTS is higher in individuals whose professional activity involves repetitive movements or wrist overload. The normal tissues pressure in the carpal tunnel region is 25 mmHg and a maximum of 32 mmHg during carpal flexion. In patients with CTS, the tissue pressure may be as high as 110 mmHg during flexion and 90 mmHg at wrist extension^(3)^.

The CTS diagnosis is essentially clinical, and the history and physical examination have 94% sensitivity^(4)^. Clinical findings of the disease include pain and dysesthesia in the median nerve distribution, in the region of the thumb, index, middle lateral half of the ring finger, which may worsen at night. The physical examination comprises the classical Tinel's and Phalen's tests for the disease diagnosis. The Tinel's test is performed by percussing over the median nerve region, which results in dysesthesia in the region of this nerve. In the Phalen's test, the symptoms are triggered by full palmar flexion of the wrists during 30 to 60 seconds, causing the typical pain^(5)^.

As regards complementary imaging methods, electroneuromyography is most frequently used to confirm the diagnosis, representing the gold standard to measure the median nerve latency with a good accuracy to detect CTS. However, false negative results are observed in 10% to 15% of cases^(6,7)^, particularly because of variation in the normal innervation pattern^(8)^. The methods specificity is reported to be as high as 95% to 99%. Some studies show that, in asymptomatic control groups, older patients may present with longer conduction times and false positive results may occur. In a study with 125 asymptomatic individuals, electrophysiological neuropathy was observed in 18% of the sample^(1)^.

Ultrasonography (US) is an excellent complementary method to detect CTS in asymptomatic patients, and should be performed with a high-resolution apparatus coupled with high-frequency linear transducer. In some studies, US results rival those from electroneuromyography. As sonographic measurements are considered, the median nerve cross sectional area at the level of the carpal tunnel entrance, adjacent to the pisiform is one of the most useful measurements. However, the cutoff value in symptomatic patients is not unanimity in the literature, ranging between 9 and 10 mm^2(9)^, and in some studies the control group (asymptomatic patients) may present values of up to 9.6 mm^2(10)^, overlapping the values of patients with CTS. Some studies demonstrate that CTS can be ruled out in individuals with values of up to 8 mm^2^ and in those diagnosed without the help from nerve conduction studies in cases where the measurement is > 13 mm^2(9)^.

In an article recently published in **Radiologia Brasileira** by Castro et al.^(11)^, asymptomatic health professionals underwent wrist US for measurement of the median nerve area, and values ≥ 9 mm^2^ were found in 34% of the sample, in most of cases, older patients, possibly with a great number of false positive results. However, one could not rule out the hypothesis of some of those individuals actually presenting pre-clinical CTS, particularly in cases where Tinel's and Phalen's tests were positive.

In a literature review, it was possible to observe that ultrasonography is useful to confirm the presence of CTS in symptomatic patients with negative or inconclusive electroneuromyography, like in the cases of diabetic patients or those at more advanced ages, particularly those above 65 years. Additionally, US is very useful in cases where the clinical diagnosis of CTS is confirmed and the physician wants to know if there is any specific structural median nerve alteration, including tumors or pseudotumors, besides diseases extrinsic to the nerve within the carpal canal which might increase the tissue pressure in the region^(5,12)^.
